# Report of the HIMSS-SIIM Enterprise Imaging Community Data Standards Evaluation Workgroup: Anatomic Ontology Assessment

**DOI:** 10.1007/s10278-024-01118-6

**Published:** 2024-06-10

**Authors:** Dawn Cram, Monief Eid, Mitchell M. Goldburgh, Jason Nagels, Lawrence Yudkovitch, Alexander J. Towbin

**Affiliations:** 1 PaxeraHealth, 85 Wells Street, Newton, MA 02459 USA; 2 Ministry of Health, Riyadh, Saudi Arabia; 3 Industry Solution, NTT DATA Services, Plano, TX USA; 4 Nagels Consulting, Newmarket, ON Canada; 5 Cleerly, New York, NY USA; 6 Department of Radiology, Cincinnati Children’s Hospital, Cincinnati, OH USA; 7 Department of Radiology, University of Cincinnati College of Medicine, Cincinnati, OH USA

**Keywords:** Enterprise imaging, Ontology, Body part, Structured terminology, Medical imaging

## Abstract

Previously, the lack of a standard body part ontology has been identified as a critical deficiency needed to enable enterprise imaging. This whitepaper aims to provide a comprehensive assessment of anatomical ontologies with the aim of facilitating enterprise imaging. It offers an overview of the process undertaken by the Health Information Management Systems Society (HIMSS) and Society for Imaging Informatics in medicine (SIIM) Enterprise Imaging Community Data Standards Evaluation workgroup to assess the viability of existing ontologies for supporting cross-disciplinary medical imaging workflows. The report analyzes the responses received from representatives of three significant ontologies: SNOMED CT, LOINC, and ICD, and delves into their suitability for the complex landscape of enterprise imaging. It highlights the strengths and limitations of each ontology, ultimately concluding that SNOMED CT is the most viable solution for standardizing anatomy terminology across the medical imaging community.

**Supplementary Information:**

The online version contains supplementary material available at 10.1007/s10278-024-01118-6.

## Introduction

Communication in healthcare drives quality, costs, and outcomes [1]. In 2019, the Healthcare Information and Management Systems Society (HIMSS)-Society for Imaging Informatics in Medicine (SIIM) Enterprise Imaging Community (HSEIC) identified body part as a key data element needed to enable consumption and interoperability of medical imaging data [2]. While multiple anatomic ontologies exist, to this point, none have been adopted for medical imaging. Namely, there is no single ontology used within enterprise imaging software solutions and across different medical specialties that enables and supports cross-specialty imaging workflows.

A subsequent HSEIC white paper described the problem stating, “Despite the existence of mature data language and communication standards, such as LOINC, SNOMED CT, DICOM, HL7 version 2, as well as new standards like FHIR, there is limited awareness of these standards among providers at the point of data creation” [2]. As detailed in that white paper, the HSEIC determined anatomy to be the most impactful data element to enable multi-disciplinary relativity, image consumption, and cross-organizational image sharing [2].

To address this concern, the HSEIC formed the HSEIC Data Standards Evaluation workgroup (DSEWG). The workgroup was tasked with assessing existing anatomic ontologies with a goal of identifying the features of an ideal ontology and subsequently recommending those that could support the cross-specialty imaging workflows required to enable enterprise imaging.

This report summarizes the DSEWG’s collection of input from providers and industry, development of necessary criteria, and recommendations of a strategy to associate standardized anatomical terms with medical imaging studies to best support enterprise imaging.

## Background

The DSEWG’s core membership was composed of five experienced imaging informaticists [DC, ME, MMG, JN, and LY] with over 100 years of collective experience leading imaging informatics and enterprise imaging projects. Workgroup members were recruited via a general call for volunteers from the HSEIC leadership to the entire community. To be considered as a core member of DSEWG, volunteers were required to be members of the HSEIC and regularly participate in workgroup activity. Volunteers were not allowed to participate if they had current or prior experience working on behalf of or for a considered ontology. Volunteers who did not regularly participate in workgroup activity were not considered to be part of the core workgroup.

The DSEWG was tasked by the HSEIC with evaluating existing anatomy nomenclatures whether or not part of an ontology. Efforts were first focused on identifying required criteria and developing a scoring model to assess the various nomenclatures. Efforts at this point led the DSEWG to conclude that anatomy terminology without structure would not suffice and only ontologies addressing anatomy would continue to be evaluated. After the initial work was complete, the DSEWG then analyzed the ability of identified ontologies to enable imaging across the healthcare enterprise.

## Guiding Principles

Throughout the process, the DSEWG was guided by the principles of neutrality, objectivity, transparency, and inclusivity.

### Neutrality

The workgroup strived to build a scoring model that was not biased by the existing features of anatomic ontologies. To support neutrality, a consecutive task approach was employed and included:

1. Core workgroup membership was limited to subject matter experts without a current or past role with any entity being assessed.
2. Involvement of, and interviews with, representatives of entities with anatomic ontologies did not begin until the scorecard and scoring model was complete.
3. Each ontology was evaluated individually, rather than in comparison, until the gap analysis step.
4. Results of the evaluation, gap analysis, and workgroup recommendation remained confidential and limited only to core workgroup members and HSEIC leadership throughout the process and until publication.

### Objectivity

The workgroup strived to create a scorecard that would rely on quantitative data rather than subjective assessments. It was equally important the results could be easily explained to the greater community as well.

### Transparency

At the conclusion of this activity, the workgroup was charged with creating a detailed report explaining their work and describing the rationale for their final recommendation.

### Inclusivity

The workgroup sought participation from across the enterprise imaging community. Participants spanned multiple medical specialties, regions of the world, and roles including providers, imaging technology experts, and vendors.

## Goals and Methods

The workgroup strived to identify a solution that would enable global systems interoperability. For purposes of this task, systems interoperability was defined to include the following:

1. Information systems utilized by medical imaging departments (e.g., electronic medical record, radiology information system, cardiovascular information system, laboratory information system, and ophthalmology information system);
2. DICOM and non-DICOM image archives (e.g., vendor neutral archive, picture archiving, and communication systems [PACS]);
3. DICOM and non-DICOM image capture devices both (e.g., magnetic resonance imaging, optical coherence tomography, and digital single lens reflex camera);
4. Other workgroup-identified systems where interoperability challenges may impact clinical care (e.g., image post-processing systems).

Because the workgroup strived for a global solution, the ideal solution(s) would be a language agnostic, coded data set.

Upon forming, the workgroup identified five goals to be achieved.

### Goal 1: Identify Existing Standards and Anatomic Ontologies

Potential anatomic ontologies were identified via a query of HSEIC members and research by the DSEWG. Ontologies were considered if they represented a unique source of anatomic structures. Initially, a list of thirteen potential ontologies was identified (Table 1). However, upon review of the list, the DSEWG concluded that two of the identified potential ontologies, Digital Imaging and Communications in Medicine (DICOM) Part 16 and Health Level Seven (HL7), did not fit the criteria of having produced a standard anatomy ontology. Both were determined to be medical data structural and communication standards accommodating the use and exchange of standard ontologies, rather than being the source of those ontologies. Therefore, they were removed from the list of potential ontologies and the scorecard was formulated to address each ontology’s ability to be effectively transmitted via these standards.

**Table 1:** List of identified potential ontologies and their parent organization

**Identified potential ontology**	**Parent organization**
International Classification of Diseases, Version 11 (ICD-11)	The World Health Organization (WHO)
Digital Imaging and Communications in Medicine (DICOM)	National Electrical Manufacturers Association (NEMA) - Medical Imaging & Technology Alliance (MITA)
Health Level 7 (HL7)	Health Level 7 International
NYU/Mayo Dermatology Initiative	New York University Langone Health and the Mayo Clinic
A.D.A.M. Interactive Anatomy	Ebix, Inc.
ACR Common Table	American College of Radiology (ACR)
Foundational Model of Anatomy (FMA)	Structural Informatics Group, University of Washington
Logical Observation Identifiers Names and Codes (LOINC)	The Regenstrief Institute
NYU Anatomy Mapper	New York University (NYU)
Radiology Lexicon (RadLex)	The Regenstrief Institute (previously Radiologic Society of North America, RSNA; now associated with LOINC)
Systematized Nomenclature of Medicine Clinical Terms (SNOMED CT)	International Health Terminology Standards Development Organisation (IHTSDO)
Unified Medical Language System (ULMS)	The National Institutes of Health (NIH)
Visible Human Project	The National Institutes of Health (NIH)

### Goal 2: Identify Needed Characteristics and Criteria

The DSEWG began the process to identify the challenges faced by clinicians due to the lack of a standard body part ontology. The workgroup developed a survey designed to gain insight into how clinicians in various specialties interact with body part labeling of images within and external to their specialty.

The DSEWG also set out to collect feedback from diagnostic users on perceived gaps in exchanging imaging studies. Specifically, the target audiences were physicians, although the survey was distributed to a broader audience through HIMSS and SIIM membership communications.

The focus of the survey questions was on the exchange of imaging studies and where gaps in anatomical description caused the greatest challenges in the availability and accessibility of prior studies. The survey questions were reviewed and revised by the workgroup and HSEIC co-chairs before being sent to the community.

The survey contained 27 questions and was hosted by HIMSS on their survey platform (SurveyMonkey Inc, San Mateo, California) (Supplementary Table 1). Invitations were sent via email from HIMSS and SIIM employees to HSEIC members who were registered as members of their respective societies.

There were 136 respondents to the survey from across the globe: 38 from the United States (28%), 24 from Europe (18%), 26 from Canada (19%), 21 from Asia (15%), 2 from Africa (1.3%), 1 from Australia (0.7%), and 24 from the Middle East (18%). Respondents were mostly radiologists and informaticists. Other specialties represented included dermatology, cardiology, gastroenterology, emergency medicine, orthopedics, endocrinology, radiation oncology, and primary care. Non-physician roles included PACS administrators and technologists.

The survey results confirmed that there was not a consistent ontology in place. In fact, each specialty had references to one or more ontologies, limiting interoperability.

The responses illustrated a few key findings related to anatomy:

1. Ambiguity of descriptors in the clinical findings resulted in difficulty identifying the anatomy of concern and caused increased work to process outside studies for 58 of the 136 respondents (43%).
2. 64 of the 136 respondents (47%) found that outside images did not display correctly.
3. 76 of the 136 respondents (56%) shared that they did not use a standards-based description of anatomy for reporting with 31 respondents uncertain (23%).
4. In many cases, the consumer of the imaging study selected an import study description based on the written report due to ambiguity in the outside descriptor.

The DSEWG collectively determined that a key success of the provider survey was that the responses illustrated the questions were clearly understood by the respondents. Based upon the responses, it was also determined through workgroup consensus that the overall problem was validated.

Following the provider survey, the DSEWG solicited additional input from specialists through workgroup interviews and use case submissions. The imaging specialists solicited were suggested by HSEIC community members and known to been involved with enterprise imaging projects at their respective institutions. The workgroup collectively interviewed these providers:

- Two cardiologists, one from Saudi Arabia and one from India
- Two radiologists, one from the United States and one from South Africa
- One gastroenterologist from Canada
- Two dermatologists from the United States
- One ophthalmologist from the United States

Interviews were recorded as part of the overall workgroup session recordings. Interviewees were questioned by the workgroup members based upon their respective specialty. Variances in specialty imaging modalities, workflows, and extent of multispecialty relativity needs called for a few specific questions to begin and followed by a more open discussion approach. Feedback from each session was analyzed by the workgroup members for common themes and specialty-specific workflows.

### Goal 3: Develop a Scoring Model

Utilizing the information gathered through workgroup member research, the provider survey, and the imaging provider interviews, a scoring matrix was developed.

Group consensus determined that the evaluation of each anatomy ontology would be based upon human anatomy, both surface and internal. Microscopic anatomy was not included within the assessment.

The DSEWG identified multiple criteria for assessment via group discussion. These criteria were categorized within two primary sections: general characteristics and ontology sustainability. Each identified criterion focused on solutions that could be encoded into interoperability standards for DICOM, FHIR, and cross-enterprise document sharing (XDS). Criteria evaluated for each section are included in Table 2.

**Table 2:** Scoring Model used by the Data Standards Evaluation workgroup

**Section 1. General characteristics**	**Weight**	**Basis for score (detail)**
Cost to organizations Question(s): Q4–Q5	3	Free = 5|country fee = 4|regional or HIE fee = 3|vendor fee = 2|provider fee = 1 Weight = 3 due to the challenges of widespread adoption with a fee-based availability
Can terminology be transmitted via a standard coded data type such as with HL7, FHIR, DICOM or IHE XDS? Question(s): Q6–Q10	3	Single field; uses alphanumeric tags = 5|requires multiple fields or non-standard characters = 3|requires multiple fields and non-standard characters = 1 Weight = 3 because usability and interoperability would be challenging without
Multilingual Question(s): Q11–Q13	2	Coded= 5|non-coded but translation mappings available for 5 or more languages = 4|non-coded and translation mappings available for less than 5 languages = 1 Weight = 2 because multilingual becomes more important if not a coded data set. Language support is necessary for global interoperability
Hierarchical structure Question(s): Q14	3	(Yes = 5|No = 1) Weight = 3 because interoperability will be negatively impacted without a hierarchical structure
Supports anatomic variants Question(s): Q15	1	Allowed values: (Yes = 5|No = 1) Weight = 1 because support for anatomic variants is a nice to have but should not significantly impact assessment
Includes laterality Question(s): Q16	3	Laterality included = 5|separate modifier can address in conjunction with ontology = 3|ontology does not include laterality or support modifier = 1 Weight = 3 because laterality is necessary for appropriate relativity internal to specialties and across specialties. Laterality inclusion within the vocabulary supports various languages as well
Orientation included within ontology Question(s): Q17	3	For purposes of this question, orientation refers to the situational orientation of the anatomic region being imaged (e.g., distal, proximal, anterior, posterior, medial, and lateral), rather than the projection orientation of the image in relation to the patient (e.g., prone and supine) Supported = 5|not supported = 1 Weight = 3 because some specialties require anatomic labeling to be inclusive of orientation (e.g., dermatology)
Includes disorder imaged Question(s): Q18	0	e.g., wound 1, 2, or mole, or wart Included = 5|not supported = 1 Weight = 0 because would be nice to have for specialties such as wound care but does not relate to all specialties
Maps across specialties Question(s): Q19	3	Applies a crosswalk to map across specialties, within the ontology, such as use of a coding crosswalk, categories/groupers, synonyms, or, at minimum, through use of inherent terminology hierarchy (e.g., top level anatomic region/body part maintains consistency across specialties) Crosswalk exists and is maintained = 5|crosswalk exists but not maintained = 4|no crosswalk = 1 Weight = 3 because interoperability across disciplines necessitates relativity

**Section 2. Ontology sustainability**	**Weight**	**Basis for score**
Organizational structure Question(s): Q20–Q21	3	Organization is sustainable a. Organization receives consistent and adequate funding b. Organization has multiple sources of income c. Organization is International d. Organization is governed by an independent body Yes to 4 of 4 = 5|Yes to 3 of 4 = 3|1 or less = 1 Weight = 3 because the organization's sustainability speaks to the consistency and maintainability required for wide-spread adoption and reduces provider and vendor adoption risks
Vendor usage Question(s): Q22	0	Number of vendors utilizing the anatomic region vocabulary from this ontology Yes/No: If yes, reviewer to provide list of known vendors which have implemented Weight = 0 because this will be used only for informational purposes when available; availability of this information may be significantly limited
Partnerships Question(s): Q23–Q24	2	Is currently partnered with medical data structural standards such as DICOM and HL7 Yes = 5|No = 1 Weight = 2 because demonstrates adoptability
Open-source terminology (vs proprietary) Question(s): Q25	1	Is the ontology open-source vs proprietary from an intellectual property perspective? Open source = 5|proprietary = 1 Weight = 1 because if the ontology is open source, it can be managed and maintained by another organization, as it would remain part of the public domain in the event of dissolution of the ontology’s organizational. However, there is still a risk the ontology could become deprecated without any organization continuing its management and maintenance
Anatomic Region Workgroup Active Question(s): Q26–Q28	2	The ontology’s number of specialties with an active workgroup focused on anatomic region (or alternatively called body part) terminology 7 of 7 plus others = 5|7 of 7 = 4|3 of 7 = 3|2 of 7 = 2|1 of 7 = 1 Weight = 2 because demonstrates multi-disciplinary support and continuous maintenance of clinical needs The 7 specialties = radiology–cardiology–dermatology–ophthalmology–gastroenterology–surgery–pathology. These specialties were selected as an area of focus for this assessment since they provide a broad scope of modality types, DICOM and non-DICOM imaging, and utilize both encounters and orders-based imaging workflows

The third section of the scoring matrix focused on two clinical use cases. These use cases allowed the DSEWG to describe how and why images were captured to document or diagnose pathology along the patient care pathway. The detailed use cases are included as Supplementary Material 1 and 2.

In collaboration with the HSEIC BP-SITE leadership, seven imaging specialties were identified as unique paradigms crucial to assess anatomy nomenclatures. These specialties were selected as they represent various types of medical imaging, utilize both DICOM and non-DICOM acquisition devices, employ a mix of orders-based and encounters-based workflows, and encompass unique body part characteristics and relationships. Specific assessment questions were also developed to identify each nomenclature’s ability to support the imaging performed by, and consumed by, the seven specialties of focus, which included Dermatology, Cardiology, Radiology, Ophthalmology, Surgery, Gastroenterology, and Pathology.

The first use case follows the imaging continuum of a patient with a nasopharyngeal mass. This use case was derived from The Importance of Body Part Labeling to Enable Enterprise Imaging White Paper [2]. The second use case was provided by an ophthalmologist and describes the imaging continuum of a patient with a malignant primitive neuroectodermal tumor (PNET) of the optic nerve.

A scoring model was formulated to evaluate each ontology independently. Each question in the scoring model was weighted from 0 to 3.

- Weight = 0 was applied to criteria determined to be for informational purposes only
- Weight = 1 was applied to criteria determined to be nice to have
- Weight = 2 was applied to criteria determined to be moderate importance
- Weight = 3 was applied to criteria determined to be of highest importance

A basis for scoring each item was detailed, to include the grading scale for each response, with a 5 being the highest possible score and 1 being the lowest.

All workflow steps for the two use cases were incorporated within the scorecard. The model used the same two criteria for each workflow step with a possible Boolean of Yes/No only, where Yes = 5 and No = 1.

1. Expected relativity achieved
  BASIS FOR SCORING: Upon reviewing the Anatomical Region response for the patient’s current exam, the patient’s prior imaging (workflow steps preceding the current step) can appear as a relative prior based upon anatomic region labeling?<div class="NodiCopyInline">BASIS FOR SCORING: Upon reviewing the Anatomical Region response for the patient’s current exam, the patient’s prior imaging (workflow steps preceding the current step) can appear as a relative prior based upon anatomic region labeling?</div>
2. Relationship mechanism available
  BASIS FOR SCORING: Is a crosswalk mechanism in place? Mechanism = coding crosswalk, categories/groupers, synonyms, or, at minimum, through use of inherent terminology hierarchy (e.g., top-level anatomic region/body part maintains consistency across specialties; enables decisive relationships across medical imaging specialties).

A weight of 3 was assigned to most use case criteria, with exceptions for the first workflow step when relativity with previous workflow steps was either not possible or not expected. In this instance, a weight of 0 was applied. The calculation for scoring was developed to implement scaling the weight for each row by the rating, similar to a Six Sigma Pugh matrix. The weighted score for each element was determined by the following formula: weighted score = ((raw score − 1)/4) * weighting factor. The average of the weighted scores was determined for each section of the request for information (RFI) by determining the mean of all weighted scores for a section where the weighting factor was not 0. Finally, the sum of the average of the weighted scores was calculated to determine the total score for each ontology.

### Goal 4: Analyze the Existing Standards Using Scoring Model

Initially, the DSEWG had planned to independently evaluate each identified ontology. However, after the scorecard was developed, the workgroup became concerned that a deep knowledge of each ontology was essential to perform an accurate assessment. To address this concern, the workgroup developed a RFI allowing representatives from each ontology to answer the questions themselves. Thus, the DSEWG identified a representative or administrative contact for each ontology and sent the RFI via email. Each entity determined their ontology’s responding representative to complete the DSEWG survey using SurveyMonkey provided and managed by SIIM. The RFI survey questions are included within Supplementary Table 2.

### Goal 5: Identify Gaps in Existing Standards

A Gap Analysis was done to provide greater insights beyond scoring. It focused on several areas of noticeable variation, such as identified usage drivers of the specific ontology. The workgroup’s gap analysis, followed by a viability assessment serves as the basis behind the workgroup’s recommendations within this report.

## Results

### Interim Information

During the DESWG’s evaluation process, three initiatives were identified that had the potential to impact the assessment. Details of each initiative are included below.

1. **National Interim Clinical Imaging Procedure (NICIP)-United Kingdom National Health Service (NHS)**
  In 2007 the United Kingdom National Health Service (NHS) published the National Interim Clinical Imaging Procedure (NICIP) Code Set to provide “a common, consistent and unambiguous representation of imaging procedures, for consistent recording and sharing of information” [3]. The table, and its associated application programming interface (API), was updated every six months and has been distributed for mandated use by the NHS since October 2009 [4]. In 2016 the Clinical Imaging Management Group (CIMG), charged with overseeing the NICIP consulted providers and other key stakeholders to determine if it was possible to replace the NICIP with SNOMED CT, due its adoption by healthcare organizations and the impact to cross-organizational image and data exchange. Ultimately, the CIMG recommended that NICIP be deprecated, and that SNOMED CT be mandated for used by the NHS.
2. **NYU/Mayo Dermatology Initiative**
  This initiative was born from a collaboration initially formed between Dermatologists at New York University Langone Health and the Mayo Clinic. Ultimately, this work led to the development of a standard for the classification of surface anatomic location for use in clinical practice and epidemiologic research [5]. The group developed a list of coded, surface anatomy terms with static hierarchy and specific to Dermatology. Currently, the group is working with the International Classification of Diseases (ICD) for incorporation into ICD-11.
3. **Open Imaging Data Model**
  This is a structural model which utilizes existing ontologies and provides a crosswalk mechanism across various, common ontologies [6]. The project is currently in its infancy and more specific to Radiology, although it is expected to be expanded to all medical imaging specialties. At the time of publication, this initiative is in review by the American College of Radiology (ACR).

### Results of RFI

Representatives from three of the eleven ontologies responded to the RFI: SNOMED CT, Logical Observation Identifiers, Names, and Codes (LOINC), and the International Classification of Diseases (ICD). Two of the eleven ontologies no longer existed as independent entities. In 2018 RadLex merged with LOINC to create a unified nomenclature [7]. The remaining RadLex terminology supports the Radiology specialty only and was not considered to be sufficient for Enterprise Imaging. Additionally, the NYU/Mayo Dermatology Initiative was incorporated into ICD-11. The remaining six chose not to submit responses to the RFI, relaying to the DSEWG that they were either not an ontology, including both terminology and structure, or they would not meet the requirements for Enterprise Imaging [2]. The results of the RFI are included in Supplementary Table 3. After review of the completed RFI, the DSEWG asked the ontology representatives for clarification of some of their responses. This clarification occurred via written communication and video conference with the work group. Based on these responses, SNOMED CT had the highest sum of average weighted scores (10.28). This score was much higher than ICD-11’s sum of average weighted scores (4.81). Highlights of the RFI assessment are included below.

1. **LOINC Assessment**
  Although LOINC completed most of the Ontology RFI, multiple questions remained unanswered or incompletely answered. Eventually, the DSEWG determined that scoring the ontology was not possible.
  Following the DSEWG’s gap analysis, the lack of a score was determined to not be impactful to the final recommendation. The LOINC database utilizes a field named “SYSTEM,” which sometimes refers to a body part. While Radiology-specific imaging codes, and some Ophthalmology imaging codes, appear to have relevant anatomy terms, the availability of relevant anatomy terms for other imaging specialties becomes sparce and is even incoherent at times. For example, when looking at codes related to clinical photos:
  46211-9 Postoperative photo is correlated with a “SYSTEM” of “XXX” (note: “XXX” is the actual value).
  62709-1 PhenX - retinal digital photography protocol 111501 is correlated with a “SYSTEM” of “^Patient”
  72170-4 Photographic Image is correlated with a “SYSTEM” of “{setting}”
  96182-1, 96183-9, and 96184-7 are all Photographic Image codes for Plastic Surgery, Dermatology, and Wound Care respectively. All three are also correlated with a “SYSTEM” of “{setting}”
  29111-2 Photo Documentation is correlated with a “SYSTEM” of “eye.right”
  Following the viability assessment, the DSEWG unanimously agreed to eliminate LOINC, due to the following:
  - The lack of a foundational hierarchy to enable decisive relationships across medical imaging specialties
  - The current anatomic focus on radiologic anatomy
2. **ICD-11 Assessment**
  ICD-11 received the highest marks on some criteria and some use case workflow steps, except as follows:
  1. Under Section 1, General Characteristics of Ontology:
    - Supports Anatomic Variants, ICD-11 responded “No” which was a Boolean answer rendering its score a 1 out of 5. This question had a weight of 1.
    - Includes Laterality, ICD-11 responded that it utilizes modifiers to address laterality rendering its score a 3 out of 5. This question had a weight of 1.
    - Maps Across Specialties, while ICD-11 responded that a crosswalk exists inherently based upon the ontology’s hierarchy, further research by the DSEWG and ICD-11’s responses to the workflow steps within each use case, it is evident that a relationship mechanism does not exist between its surface and functional anatomy rendering its score a 1 out of 5. This question had a weight of 3.
  2. Under Section 2, Ontology Sustainability:
    - Organizational Structure, ICD-11 responded yes to 2 of 4 requirements and the DSEWG identified that due to the organization’s governing body, another requirement should be considered positively answered which rendered a score of 3 out of 5. This question had a weight of 3.
    - Partnerships, ICD-11 responded “No, is not partnered with medical data structural standards such as DICOM and HL7”, which was a Boolean answer rendering its score a 1 out of 5. This question had a weight of 2.
    - Anatomic Region Workgroup Active, ICD-11 responded that its anatomy ontology supports and has active workgroups tasked with managing and maintaining terminology for the specialties of Radiology, Cardiology, Dermatology, Ophthalmology, Gastroenterology, Surgery, and Pathology, rendering its score a 4 out of 5. This question had a weight of 2.
  3. Use-Case 1:
    No Expected Relativity Achieved and No Relationship Mechanism Available was noted by the DSEWG from workflow steps 4 through 17 and step 19.
    ICD-11’s score = 1, out of 5, for 14 of the 22 total workflow steps associated with use-case 1. Workflow step 19 had a weight of 0 as body part relativity was not required for this step.
  4. Use-Case 2:
    No Expected Relativity Achieved and No Relationship Mechanism Available was noted by the DSEWG from workflow steps 27 through 29.
    ICD-11’s score = 1, out of 5, for 3 of the 7 total workflow steps associated with use-case 2.
    ICD-11 provides an anatomy broken down into three hierarchies, Functional Anatomy, Surface Topography, and Partonomic View. However, there is no indication of a mapping architecture across these three hierarchies, nor any clear foundational architecture to support future development. Although ICD representatives had responded to other questions throughout the process no clarification was received for additional questioning related to mapping across the three classifications. The DSEWG determined that enterprise Imaging relativity would not be achievable without the ability to map across Functional and Surface anatomy, at minimum. Following the viability assessment, the DSEWG realized that further clarification of ICD-11’s mapping across classification would not impact the final recommendation.
    While the Health Data Standards Crosswalk of the Global Digital Health Partnership White Paper on Interoperability [8] indicates that ICD is used in 21 countries, only ICD-9 and ICD-10 are formally implemented. A deadline for ICD-11 implementation has not been established and according to the World Health Organization “64 Member States are in different stages of ICD-11 implementation” [9].
    **3. SNOMED CT Assessment**
    SNOMED CT received the highest possible marks for almost every criterion and use-case workflow step. It received lower than the highest potential score for only four questions. These questions are:
    1. Under Section 1, General Characteristics of Ontology: Cost to Organizations, SNOMED scored a 4 out of 5 since there is, at least impact, a country fee. This question had a weight of 3.
    2. Under Section 2, Ontology Sustainability: Open-source terminology (vs proprietary), SNOMED responded “No” which was a Boolean answer rendering its score a 1 out of 5. However, the DSEWG deemed this would have little to no impact to its sustainability per SNOMED’s further explanation stating, “Due to the nature of its use in patient healthcare records globally, the ontology would be managed and maintained by another organization, inheriting the exiting intellectual property.” This question had a weight of 1.
    3. Use-Case 1 (2 questions):
      No Expected Relativity Achieved and No Relationship Mechanism Available was noted by the DSEWG for workflow steps 21 and 22.
      SNOMED CT’s score = 1, out of 5, for 2 of the 22 total workflow steps associated with use-case 1. This question had a weight of 0 as body part relativity was not required for these steps.
      Of note, SNOMED CT is already used within medical imaging for anatomic classification including:
      1. HL7’s Fast Healthcare Interoperability Resources – the BodyLocationVS utilizes SNOMED CT codes exclusively [10].
      2. DICOM’s preferred anatomy ontology is SNOMED CT [11].
      3. The United Kingdom’s National Health Service has moved from its own NICIP to SNOMED CT exclusively, for medical imaging anatomy.
    Additionally, SNOMED CT has multiple resources and tools to assist the developer community including the SNOMED CT Authoring Platform and the Reference Set & Translation tool. Available open-source tools such as Snow Owl®, a collaborative terminology browsing and authoring platform built on the open-source International Health Terminology Standards Development Organisation Terminology Server, and the official Snow Owl^®^ RESTful API offer robust mechanisms to incorporate SNOMED and its terminology relationships within software.

### Assessment Gap Analysis

Table 3 details how each of the three final ontology candidates performed in comparison on gap points noted with any of the three.

**Table 3:** Gap analysis comparison of the three final ontology candidates

	**LOINC**	**ICD-11**	**SNOMED CT**
**Relationships across medical imaging specialties**	Radiology-specific structure and vocabulary for anatomy	Major gap between functional and surface anatomy	Complex relationships across all supported imaging specialties
**Drivers of anatomic region determination**	Anatomy determination driven by body part/region being imaged (procedure code for orders-based imaging)	Anatomy determination driven by imaging result (diagnosis) and billing (area of pathology)	Anatomy determination driven by body part/region being imaged (procedure code for orders-based imaging) and separately reason for exam
**Current global adoption**	Used globally (10)	Not used globally (10)	Used globally (10)
**Cost**	Free	Free	Not free, although free for anyone, including institutions, within member countries
**Partnerships**	SNOMED, HL7	Not partnered with medical data standards (although is a World Health Organization initiative)	Partnered with HL7, DICOM, LOINC, and others
**Supported medical specialties**	Narrow	Medium	Broad

### Viability Assessment

Following the gap analysis, the DSEWG determined that a viability assessment could further aide in determining if additional information was necessary from any of the three ontologies prior to providing its recommendation. For purposes of this assessment, viability was determined by whether the ontology sufficiently addressed the broad scope of anatomic terms and terminology relationship complexities to support Enterprise Imaging by addressing two primary questions.

Question 1: Is the [Ontology]’s anatomy terminology viable for Enterprise Imaging Workflows (order through interpretation workflow steps)?

Question 2: Is the [Ontology]’s anatomy terminology viable for Enterprise Imaging Exam Relativity (data mining, data migrations, hanging protocols, relevant priors, etc.)?

The DSEWG conducted the viability analysis. This activity was conducted as a group exercise designed to identify deficiencies that would preclude the use of the ontology in enterprise imaging workflows. The DSEWG identified the following primary deficiencies of each ontology. The list of all deficiencies are included in Table 4.

1. ICD: Anatomy is determined based upon the result or diagnosis of the imaging [8]. This does not align with the need for a standard anatomy ontology that supports identification before or during image acquisition.
2. LOINC: Anatomic terms are based on radiologic anatomy. Comprehensive term for surface anatomy were not present.
3. SNOMED: The ontology is not free to use in all countries.

**Table 4:** Ontology viability assessment for use in enterprise imaging

Ontology	Viable: Yes/No	Why	Why not
LOINC	No		Radiology-specific (RadLex dictionary) No anatomy mapping across imaging specialties and/or between surface and internal anatomy Note: may require SNOMED CT to achieve cross-discipline relativity
ICD-11	No		Major gap between surface and functional anatomy and no true hierarchical approach ICD-11 determines anatomy by diagnosis/pathology (result of the imaging)
SNOMED CT	Yes	Broad medical specialty support Global presence Anatomy determination driven by body part/region being imaged (procedure code for orders-based imaging) and separately reason for exam	Although not free to all, it is free to many (member countries) *Availability of the SNOMED CT Authoring Platform and the Reference Set & Translation tool may be an area of further discussion

The DSEWG determined that the listed deficiencies of ICD-11 and LOINC made the ontologies non-viable for enterprise imaging purposes. The deficiency of SNOMED CT had the potential to limit world-wide adoption but did not preclude its use for enterprise imaging.

## Limitations

While the DSEWG attempted to provide an assessment of body part ontologies for enterprise imaging, there were several limitations of the effort. First, the DSEWG relied on responses to an RFI to guide their data collection and assessment. The workgroup chose this approach rather than directly evaluating each ontology independently. We believe that this approach was prudent as it allowed each ontology to represent themselves while answering the DSEWG RFI. Specifically, the DSEWG recognized that the representatives of each ontology had the most expertise regarding its strengths and limitations. As such, they would be best positioned to accurately answer detailed questions and help to determine fit with the identified system requirements. This approach proved to be beneficial as several ontology representatives identified barriers that would preclude their use for the enterprise imaging use case. While it is possible that some ontology representatives misinterpreted the system requirements, use cases, or rationale for the questions being asked, the DSEWG tried to limit this potential by providing a rationale for the ontology representatives and hosting online meetings to ask additional questions. Ultimately, we believe that this approach was the most efficient and accurate as ontology representatives are the most knowledgeable sources of information related to their product.

A second limitation of this report is the lack of statistics related to survey respondents. The survey was sent via email blast to HIMSS, SIIM, and HSEIC members. Because this message was sent in multiple across different marketing platforms (email and social media), user bases, and as a part of varying other societal messaging, the data related to the number of people who received the survey is not able to be reported.

Third, not every specialty was included in the use cases. After consultation with HIMSS, SIIM, and HSEIC leadership, the DSEWG limited the length of the use cases to promote a better response rate from ontologies. While some specialties are missing, we believe that the included use cases highlight differences in anatomy and anatomic labelling across specialties. Specifically, the use cases balanced the need for general anatomic terms (such as in radiology), focused terms (such as in ophthalmology), surface anatomy (such as in dermatology), and internal anatomy (such as is pathology).

Finally, the ontologies did not all respond to every RFI question. While this impacted some of the information collected and the ability to completely score each RFI, the DSEWG did not believe that it affected their ability to determine whether an ontology was viable to be used as part of an enterprise imaging workflow.

## Final Recommendation

Based upon the clear need for industry adoption of a single standard anatomy ontology, it is the conclusion of the DSEWG that SNOMED CT is the only viable solution. **The DSEWG recommends that the industry focus its efforts on the adoption and implementation of SNOMED CT for use in enterprise imaging**.

Furthermore, **the DSEWG encourages the HSEIC to establish a relationship with SNOMED CT to enable the community’s feedback**. The DSEWG notes that SNOMED CT has already established organizational infrastructure to support this type of relationship through their Clinical Reference Groups (CRG). Thus, the workgroup recommends establishing an Enterprise Imaging CRG. This CRG should initially focus on anatomy terms and later spearhead other data initiatives. As described by SNOMED CT in response to the DSEWG RFI:

*“Clinical Reference Groups (CRG) provide a framework to facilitate discussion between clinicians focused on specific clinical specialties or topic areas, and dialogue between clinicians and SNOMED International.”* [12].

**The DSEWG recognizes that the SNOMED CT freeset of coded concepts in the DICOM standard is not sufficient for Enterprise Imaging. The freeset is a limited subset of anatomical terms which are provided to DICOM for open usage within the standard.** Additionally, these terms represent a limited set of static codes and descriptions and do not include SNOMED CT’s inherent relationships and hierarchy necessary to establish the relativity required for meaningful Enterprise Imaging. Although the DICOM standard can support the full array of codes and relationships, for global adoption, the limited freeset would not suffice.

Finally, to ensure that the SNOMED CT works for every specialty, **the DSEWG recommends that SNOMED CT consider incorporating any surface anatomy terminology and relationship gaps identified through a comparison with the NYU/Mayo Dermatology initiative**.

It is further recommended that future endeavors look at building upon the inherent loop between SNOMED for clinical care and ICD for results, such as the map provided by SNOMED international.

*“The purpose of the SNOMED CT to ICD-10-CM map (herein referred to as “the Map”) is to support semi-automated generation of ICD-10-CM codes from clinical data encoded in SNOMED CT for reimbursement and statistical purposes”* [13].

*“SNOMED International distributes a map from SNOMED CT to ICD-10. This supports the generation of ICD-10 classified data from data originally recorded using SNOMED CT, or later mapped to SNOMED CT*” [13].

This loop can optimize the accessibility of a patient’s holistic medical imaging record by disease process for medical providers necessitating such a view. This may also enhance interpretations and AI and machine learning processes and algorithm development.

**Education of the community, both vendors and providers, will be crucial for successful SNOMED CT implementation and adoption.** It is the opinion of the DSEWG that vendors and providers alike generally have a limited understanding of the SNOMED CT anatomy ontology, resulting in its lack of use or misuse. The DSEWG believes that if the HSEIC establishes a collaborative relationship with SNOMED, imaging informaticists will be able to better understand the ontology leading to its optimal use globally. Thus, the DSEWG recommends that the HSEIC support the educational efforts needed to effectively guide the community in understanding, implementing, and adopting these recommendations.

## Conclusion

Since the advent of Picture Archiving and Communication Systems (PACS) in the 1990’s, provider organizations have struggled with inconsistent and disjointed body part terminology associated with Radiology imaging exams. This has created significant workflow challenges across clinical systems and has been exacerbated with the introduction of Enterprise Imaging concepts and specialties. The problem has been compounded by several anatomy ontologies surfacing over the past decades, various groups working on mechanisms and vocabularies in silos specific to their respective imaging specialties, and organizations resorting to establishing their own anatomy vocabularies.

If the global medical imaging community standardizes to a single anatomy ontology, notable improvements may be achieved including:

- Clinical workflows
  - Viewing/display protocols
  - Relevant comparison studies
  - Cross-specialty relationships
  - Following of clinical analysis for pathologies and abnormalities
  - Accessibility of images across medical specialties
- Data normalization/cleansing
- Image exchange
  - Such as the Integrating Healthcare Enterprise (IHE) Import and Display of External Priors (IDEP)
- Data mining
  - Analytics
  - Machine learning
- Artificial Intelligence (AI) analysis
  - Streamlining the program (software) learning process
  - Incorporation of biomarkers

The viability and sustainability of an Ontology is key to successful adoption. It is the position of the DSEWG that SNOMED CT provides the only viable solution for anatomy labeling in enterprise imaging. Several key factors supporting this claim include:

1. It provides the most comprehensive solution, with both lineal and collateral relationships supported across surface and internal (functional) anatomic structures. The complex relationship support can offer providers the ability to establish relationships across imaging exams based upon clinical need, and as highly aggregated or granular required to support those needs.
2. Anatomic region sequence is determined by the body part being imaged. Primary Anatomic Structure Sequence is driven by reason for exam. By combining drivers of body part being imaged with the reason for exam, SNOMED CT offers the ability to extend relativity to disease processes enabling a more holistic approach in Enterprise Imaging. This was a significant differentiator during the DSEWG’s viability assessment.
3. It features a coded data set, which supports ease of language extensibility for global adoption. Rather than specific English terms which would require mapped translations, unique codes are employed, and the code description is translated across languages, allowing imaging performed in one country to be relative to imaging performed in another country.
4. Its anatomy terminology encompasses many medical specialties which are crucial to Enterprise Imaging.
5. Collaboration with the HSEIC SNOMED CT will be required for subsequent education, implementation, adoption, and maintenance efforts.

The primary gap is the availability of the SNOMED CT anatomy code set, beyond its limited freeset, and programmatic relationship tools for all users, which includes the SNOMED CT Authoring Platform and the Reference Set & Translation tool. Although the GPS (Global Patient Set) is free for all to use, it is incomplete and requires significant effort to implement within medical software. Providing the relationships and hierarchy for SNOMED CT’s anatomy ontology at no cost, regardless of country membership, is critical to support its global, Enterprise Imaging adoption.

If the DSEWG recommendations are adopted, the HSEIC will need to establish an education initiative helping the community to implement and utilize the SNOMED CT Authoring Platform, Reference Set, and Translation tools. Standardizing usage of this tool and offering provider guidance on best practices should be considered as well to support ease of provider adoption.

## Supplementary Information

Below is the link to the electronic supplementary material.

Supplementary file1 (DOCX 14 KB)

Supplementary file2 (DOCX 13 KB)

Supplementary file3 (DOCX 31 KB)

Supplementary file4 (DOCX 51 KB)

Supplementary file5 (DOCX 29 KB)

## Abbreviations

ACR: American College of Radiology
AI: Artificial Intelligence
API: Application programming interface
CIMG: Clinical Imaging Management Group
CRG: Clinical Reference Groups
DICOM: Digital Imaging and Communications in Medicine
DSEWG: Data Standards Evaluation workgroup
FHIR: Fast Healthcare Interoperability Resources
HIMSS: Health Information Management Systems Society
HSEIC: HIMSS/SIIM Enterprise Imaging Community
HL7: Health Level Seven
ICD: International Classification of Diseases
IDEP: Import and Display of External Priors
IHE: Integrating Healthcare Enterprise
LOINC: Logical Observation Identifiers, Names, and Codes
NHS: National Health Service
NICIP: National Interim Clinical Imaging Procedure
NYU: New York University
PACS: Picture Archiving and Communication Systems
RESTful: Representational State Transfer
RFI: Request for information
SIIM: Society for Imaging Informatics in medicine
SNOMED CT: Systematized Nomencla
XDS: Cross-enterprise document sharing
