# Supplementary material for: Report of the HIMSS-SIIM Enterprise Imaging Community Data Standards Evaluation Workgroup: Anatomic Ontology Assessment
Source: J Imaging Inform Med. 2024 Jun 10;37(6):2709–21. doi: 10.1007/s10278-024-01118-6 (PMC11612098; doi:10.1007/s10278-024-01118-6)
Supplement: Supplementary file 1 — Supplementary file1 (DOCX 14 KB) [file 10278_2024_1118_MOESM1_ESM.docx]

Supplementary Material 1: Example of clinical use case designed to evaluate the different body part ontologies

Consider a patient with a right-sided nasopharyngeal mass. She initially presents to her family medicine physician who examines the mass and takes a photograph using a personal smartphone. Based on the patient's history of Epstein-Barr virus and allergic rhinitis, the physician’s differential diagnosis includes nasal polyps and nasopharyngeal carcinoma. Because of the differential diagnosis, the physician refers the patient to an otolaryngologist for further evaluation. The patient's appointment is scheduled for three weeks hence. In the interim, the mass enlarges and begins to bleed. Concerned, the patient takes a picture herself with her smartphone and emails it to her doctor. The family medicine physician orders an outpatient MRI to evaluate the mass. A Neuroradiologist makes a presumptive diagnosis of nasopharyngeal carcinoma. After waiting for three weeks, the patient sees the otolaryngologist in a private, office-based practice. This otolaryngologist is also concerned that the patient has a nasopharyngeal carcinoma and performs office nasolaryngoscopy. Several pictures are acquired, and a biopsy performed. The patient is told to expect minimal bleeding and to call the office if she is concerned. That evening, the packing material becomes soaked with blood and falls out of the nasal cavity. The patient takes a photograph of the packing with her smartphone and emails it to her otolaryngologist. The otolaryngologist reviews the image and recommends that the patient 5 goes to the emergency department of a local hospital for further evaluation. In the emergency department, the nurse uses a shared departmental smart device to take a photograph of the nasal cavity and shows it to the emergency department physician. After review, the emergency department physician requests that the photo be sent to the on-call otolaryngologist (from a different, hospital-based practice than the first otolaryngologist) and asks the consulting otolaryngologist to see the patient emergently. Once in the emergency room, the on-call otolaryngologist performs another nasolaryngoscopic examination, takes photographs, then cauterizes a bleeding vessel. The patient is discharged to recuperate. Meanwhile, the specimen from the earlier biopsy is sent to the pathology department where the pathologist obtains gross specimen photographs using a digital single-lens reflex (dSLR) camera and submits the tissue for fixing, embedding, and staining followed by digital whole slide scanning. The pathologist evaluates the tissue using a virtual microscopy application and signs out the case as nasopharyngeal carcinoma. With the malignant diagnosis confirmed, the original otolaryngologist takes over the care of the patient and orders a chest CT and a whole-body PET/CT to evaluate the disease extent. Imaging is performed at a different outpatient imaging facility than the original MRI. The nuclear medicine physician reports regional lymph node involvement. The otolaryngologist refers the patient to an oncologist to coordinate further care. The oncologist recommends that the patient undergo radiation therapy and chemotherapy and refers her to a radiation oncologist. The radiation oncologist obtains CT images for initial treatment planning and at subsequent visits. At predefined intervals, repeat PET/CT and MRI are also performed to monitor the patient's response to therapy. These follow-up scans reveal residual tumor in the neck lymph nodes. The otolaryngologist is informed of the findings and performs a radical lymph node dissection. During surgery, multiple photographs and brief videos are recorded using a dSLR camera. The lymph node specimens are sent for histopathology, and gross photographs and whole slide scans are acquired. Meanwhile, the patient is seen in the wound care clinic several times to ensure that the neck incision healed completely after the extensive surgery and radiation therapy. Photographic documentation is performed during each visit to monitor healing. All the images in this scenario were obtained over six months. During that time, the patient received medical attention for other chronic medical conditions as well as routine screening studies such as mammography and dermatologic mole mapping. After the lymph node resection, the patient noticed swelling in her right breast. When she mentioned the swelling at the screening mammography visit, the technologist took a photograph of the swelling along with the digital mammograms and converted the study to a diagnostic mammogram. A digital breast tomosynthesis study was obtained and the breast imager performed a targeted ultrasound. During the scenario, at least 23 imaging studies were performed by eight specialties across six services. The imaging studies and the facilities, specialties, and departments performing the imaging study are highlighted in Supplementary Table 3.
