# Supplementary material for: Report of the HIMSS-SIIM Enterprise Imaging Community Data Standards Evaluation Workgroup: Anatomic Ontology Assessment
Source: J Imaging Inform Med. 2024 Jun 10;37(6):2709–21. doi: 10.1007/s10278-024-01118-6 (PMC11612098; doi:10.1007/s10278-024-01118-6)
Supplement: Supplementary file 2 — Supplementary file2 (DOCX 13 KB) [file 10278_2024_1118_MOESM2_ESM.docx]

Supplemental material 2: Second example of clinical use case designed to evaluate the different body part ontologies

A 21-month-old female presented with a new onset squint to an ophthalmologist, who confirmed loss of vision in the affected eye. There were no features of neurofibromatosis type 1 Orbital ultrasound showed a mass posterior to the left eye. The patient was then referred for a CT of the brain and orbits for better evaluation of the mass. The CT Brain and Orbits showed a contrast-enhancing enlarged left optic nerve with no other lesions in the brain or right orbit. The features were suspicious for an optic nerve glioma and further imaging with MRI was suggested. MRI showed a soft tissue lobulated lesion in the intra-Conal optic nerve measuring 1.6 x 0.9 cm. This mass was isointense on T1 and T2, mildly hyperintense on STIR with no restricted diffusion on DWI. The mass extended to involve the left optic disk and retina and the optic nerve canal. No extension into the left cavernous sinus or intracranially was noted. The mass and the optic nerve sheath enhanced heterogeneously post contrast. The features were considered typical of an optic nerve glioma. The patient then had a left orbital enucleation. The histology findings were of malignant central nervous system Primitive Neuroectodermal Tumor (PNET) of the optic nerve, WHO grade IV which is very rare to involve the optic nerve. The patient completed chemotherapy and due to the high grade PNET, a follow up MRI brain, orbit and spine follow-up was done performed in 6 months. The follow -up MRI’s showed no evidence of recurrence in the orbits or metastasis in the brain and spine. The imaging studies and the facilities, specialties, and departments performing the imaging study are highlighted in
