# Supplementary material for: Report of the HIMSS-SIIM Enterprise Imaging Community Data Standards Evaluation Workgroup: Anatomic Ontology Assessment
Source: J Imaging Inform Med. 2024 Jun 10;37(6):2709–21. doi: 10.1007/s10278-024-01118-6 (PMC11612098; doi:10.1007/s10278-024-01118-6)
Supplement: Supplementary file 3 — Supplementary file3 (DOCX 31 KB) [file 10278_2024_1118_MOESM3_ESM.docx]

| Provider Survey |
| --- |
| 1. What image capture devices do you use? |
| 1. What types of anatomy do you capture, and do you label the images? Internal Anatomy_/ Surface Anatomy |
| 1. How often do you describe anatomy on images? |
| 1. How often do you describe anatomy on reports? |
| 1. How often to do you use a standardized nomenclature for describing Anatomy? |
| 1. How often to do you use a standardized nomenclature for describing Color? |
| 1. How often to do you use a standardized nomenclature for describing Laterality? |
| 1. How often to do you use a standardized nomenclature for describing Measurements? |
| 1. How often to do you use a standardized nomenclature for describing Range of Motion? |
| 1. How often to do you use a standardized nomenclature for describing Results Criticality and Follow Up? |
| 1. How often to do you use a standardized nomenclature for describing Severity of Findings? |
| 1. How often to do you use a standardized nomenclature for describing Shape? |
| 1. How often to do you use a standardized nomenclature for describing Staging/Grading (e.g., tumor, stenosis)? |
| 1. In your practice, is there a source or ontology that you are using for labeling your images/studies? |
| 1. How structured are your reports? |
| 1. What images do you use in your practice? |
| 1. When viewing reports from outside your practice, do you receive (check all that apply): |
| 1. Which of the following are challenges when using reports/images from outside your practice? |
| 1. Does your department create or use analytics? |
| 1. What sort of terminology challenges are you facing? |
| 1. Does your department create or use population health statistics? |
| 1. What sort of terminology challenges are you facing? |
| 1. How would you describe your role? |
| 1. What is your specialty? |
| 1. How long have you been in practice? |
| 1. How do you view images? |
| 1. Where are you located? |

Supplementary Table 1: Questions included in the provider survey
