# Supplementary material for: Report of the HIMSS-SIIM Enterprise Imaging Community Data Standards Evaluation Workgroup: Anatomic Ontology Assessment
Source: J Imaging Inform Med. 2024 Jun 10;37(6):2709–21. doi: 10.1007/s10278-024-01118-6 (PMC11612098; doi:10.1007/s10278-024-01118-6)
Supplement: Supplementary file 4 — Supplementary file4 (DOCX 51 KB) [file 10278_2024_1118_MOESM4_ESM.docx]

Supplementary Table 2: Ontology Request for Information

Questionnaire Introduction and Explanation

Despite the existence of mature data standards for clinical data, there still exists an unaddressed need for medical vocabulary standardization. Adoption of standard medical terminology for clinical systems is critical to improve interoperability, patient safety, and clinical outcomes and will allow medical practitioners to leverage the promises of precision medicine.

The following Ontology Request for Information has been designed by a collaborative HIMSS-SIIM Workgroup to speciﬁcally address the need for a standard, anatomic region nomenclature based on the applicability, relevancy, and governance of present-day medical ontologies. The Questionnaire features three (3) sections detailing key areas where ontologies will be scored. Please ﬁll out this Questionnaire to the best of your ontology's capability; answers that are either missing or incomplete will impact scoring during the ﬁnal reporting process.

The Ontology Assessment Questionnaire is broken into three critical sections, including:

**Section 1 |** General Characteristics of Nomenclature (est. 15 minutes for completion)

**Section 2 |** Nomenclature Sustainability (est. 15 minutes for completion)

**Section 3 |** Use Cases (est. 30 minutes for completion)

Due to the interconnected nature of the questions, we recommend that you plan to complete this assessment in one sitting. That being said you may click "Next" at the bottom of the screen for your answers to be saved.

*** 1. Please ﬁll out the below contact information so that our team may contact you should we have any questions.**

Name

Company

State/Province

Country

Email Address

Phone Number

*** 2. For which ontology are you ﬁlling out the following assessment?**

**3. Please provide a website link for the ontology for which you are ﬁlling out the following assessment.**

**SECTION 1 |** General Characteristics of Nomenclature

**4. Is there a fee, or other costs, to access or use your ontology’s terminology?**

No, there is No Fee

Yes, Country Fee

Yes, Regional or Health Information Exchange (HIE) Fee

Yes, Vendor Fee

Yes, Provider Fee

**5. Based on your previous answer, please identify the dollar value of the fee to access or use your ontology’s terminology. Responses are requested in United States dollars, however all answers will** be accepted.

Country Fee

Regional or Health Information Exchange (HIE) Fee

Vendor Fee

Provider Fee

**6. Can your ontology’s terminology be transmitted via a standard coded data type, such as with HL7, FHIR, DICOM or IHE XDS?**

No

Yes

*** 7. Does your ontology's terminology use characters other than alpha-numeric characters? For purposes of this question, alphanumeric characters are those comprised of the combined set of the English 26 alphabetic characters, A to Z, and the 10 Arabic numerals, 0 to 9.**

No, it does not.

Yes, non-alpha-numeric characters are required.

Yes, non-alpha-numeric characters are supported but not required.

**8. How many separate ﬁelds (data points) does your ontology’s anatomic region (body part) nomenclature utilize?**

Provide count of all available ﬁelds.

Please list all ﬁelds with short descriptions.

**9. How many separate ﬁelds (data points) are required for your ontology’s anatomic region (body part) nomenclature?**

Provide count of all required ﬁelds.

Please list all ﬁelds with short descriptions.

**10. Please provide a sample of your ontology’s encoding for the distal phalanx of the left small ﬁnger (or closest equivalent), including subregions, laterality, variants, orientation, and other applicable attributes.**

**11. Are multiple languages supported?**

Yes

No (Please Provide Nomenclature's Language)

***12. Please list nomenclature’s primary language**.

***13. Please list all additional nomenclature languages mapped by ontology.**

**14. Does your Ontology’s nomenclature make use of a hierarchical structure? For example, anatomic region maps to multiple subregions, such as shoulder, wrist, elbow mapping to upper extremity, etc.**

No

Yes

**15. Are anatomical variants supported? (e.g. six (6) toes)**

No

Yes

**16. Does your Ontology’s nomenclature include laterality or support laterality modiﬁers? Please select all that apply.**

No, does not include laterality and does not support laterality modiﬁers. Yes, laterality included within coded values.

Yes, laterality included within terminology.

Yes, separate modiﬁer is supported in conjunction with nomenclature.

**17. Does your Ontology’s nomenclature include orientation?**

**For purposes of this question, orientation refers to the situational orientation of the anatomic region being imaged (e.g. distal, proximal, anterior, posterior, medial, lateral), rather than the projection orientation of the image in relation to the patient (e.g. prone, supine).**

No

Yes

**18. Does your Ontology’s nomenclature include the disorder imaged? (e.g. Wound 1, Wound 2, Mole, Wart, etc.)**

No

Yes

**19. Does your Ontology’s nomenclature map across imaging specialties and/or between surface and internal anatomy?**

**For this question, we are looking for the application of a crosswalk to map anatomic terminology across various specialties within an Ontology, such as use of a coding crosswalk, categories/groupers, synonyms, or inherently through use of terminology’s hierarchy (e.g. same anatomic region across surface and internal anatomy which contains subregions detailed to support various specialties).**

No, a crosswalk does not exist.

Yes, a crosswalk exists and is maintained through routine reviews and updates when applicable.

Yes, a crosswalk exists but is not maintained through routine reviews and updates when applicable.

Yes, a crosswalk exists inherently based upon nomenclature’s hierarchy.

**Section 2 |** Nomenclature Sustainability

**20. How is your organization structured?**

No formal organizational structure exists.

Organization receives consistent and adequate funding.

Organization has multiple sources of income.

Organization is international.

Organization is governed by an independent body.

**21. Based on your previous answer, please provide additional information on the sources of funding, a list of all sources of income, a list of countries your organization supports or operates in, and/or the name of the independent body.**

Organization receives consistent and adequate funding.

Organization has multiple sources of income.

Organization is international.

Organization is governed by an independent body.

**22. Is your organization’s nomenclature utilized by medical imaging software vendors?**

No

Yes (Please List all Known Medical Imaging Software Vendors)

**23. Is your organization’s nomenclature utilized by EMR vendors?**

No

Yes (Please List all Known Medical Imaging Software Vendors)

**24. Has your organization partnered with medical data structural standards such as DICOM and HL7?**

No formal partnerships exist or have ever been formed.

No formal partnerships currently exist, although organization has previously partnered with data standards organizations.

Yes, formal partnerships currently exist.

**25. Based on your previous answer, please provide the following information:**

**List all standard(s) organizations and provide an explanation as to why partnership(s) was/were dissolved.**

**List all standards organizations currently partnered with your organization.**

No formal partnerships currently exist, although organization has previously partnered with data standards organizations.

Yes, formal partnerships current exist.

**26. In the event of your organization’s dissolution, would your ontology and anatomic region (body part) nomenclature be part of the public domain?**

No, both are proprietary from an intellectual property perspective.

Yes, in the event of organization dissolution, our ontology and anatomic region (body part) can be managed and maintained by another organization or entity.

Other (please provide a detailed explanation)

**27. Which medical imaging specialties does your organization’s anatomic region nomenclature support? Please select all that apply.**

Radiology

Cardiology

Dermatology

Ophthalmology

Gastroenterology

Surgery

Pathology

Other (please provide a list of all additional medical imaging specialties)

**28. Of the medical imaging specialties listed above, for which specialties does your organization have an active workgroup tasked with managing and maintaining the anatomic region nomenclature? Please select all that apply.**

Radiology

Cardiology

Dermatology

Ophthalmology

Gastroenterology

Surgery

Pathology

Other (please provide a list of all additional medical imaging specialties)

**Section 3 |** Introduction and PDF Copy of Chart

**The following table highlights diﬀerent imaging studies for patients associated with several use cases. For each imaging study detailed, the following DICOM data elements are pre-populated: Modality, Study Description, Institutional Department Type Code Sequence.**

**Please ﬁll out the Numeral-Alpha Questions as identiﬁed by the bolded letters within the chart for each of the below-listed 29 image acquisition points. This question continues over 9 parts for ease of access and review.**

**29.a Anatomic Region Sequence**

**29.b Image Laterality**

**29.c Primary Anatomic Structure Sequence**

|  | Workﬂow Step | DICOM  Modality **or** Diagnostic Service Section ID | | Study Description **or** Universal Service ID Text | | Institutional Department Type Code Sequence **or** Performing Department | Anatomic Region Sequence **or** Body Part | Image Laterality **or** Laterality Modiﬁer (N/A if Not Applicable) | Primary Anatomic Structure Sequence **or** Primary Anatomy  Mapped To |
| --- | --- | --- | --- | --- | --- | --- | --- | --- | --- |
| 1 | Family medicine physician-acquired photograph of right  sided nasal mass. | | XC | | Photo - Head & Neck | Primary Care Department | **1.a** | **1.b** | **1.c** |
| 2 | Patient-acquired  photograph of right sided nasal mass. | | XC | | Photo - Head & Neck | Patient generated | **2.a** | **2.b** | **2.c** |
| 3 | MRI nasal polyps, right-sided nasopharyngeal mass, r/o nasopharyngeal  carcinoma | | MR | | MR MR Neck w and wo IV Contrast | Radiology | **3.a** | **3.b** | **3.c** |
| 4 | Nasolarvngoscope photograph | | ES | | Nasolaryngoscope | Otorhinolaryngology | **4.a** | **4.b** | **4.c** |
| 5 | Patient-acquired photograph of packing material | | XC | | Photo - Head & Neck | Patient generated | **5.a** | **5.b** | **5.c** |
| 6 | Nurse-acquired photograph of bleeding, right sided nasal mass | | XC | | Photo - Head & Neck | Accident and Emergency | **6.a** | **6.b** | **6.c** |
| 7 | Gross specimen- intake  workﬂow right sided nasop,haryngeal  mass | | XC or GM | | Gross Specimen - Head and Neck | Pathology | **7.a** | **7.b** | **7.c** |
| 8 | Pathology slide scanning workﬂow, right-sided nasopharyngeal  mass | | SM | | Whole Slide - Head and Neck | Histopathology | **8.a** | **8.b** | **8.c** |
| 9 | CT nasopharyngeal  carcinoma, r/o metastasis | | CT | | CT Chest wo IV Contrast | Radiology | **9.a** | **9.b** | **9.c** |
| 10 | PET/CT nasopharyngeal carcinoma, r/o metastasis | | PT, CT | | PT/CT  Tumor Localization Whole Body | Nuclear Medicine | **10.a** | **10.b** | **10.c** |
| 11 | Radiation Oncology Planning  CT nasopharyngeal carcinoma, lymph node  metastasis | | RTPLAN | | CT  Treatment Planning Complex | Radiotherapy | **11.a** | **11.b** | **11.c** |
| 12 | PET/CT nasopharyngeal carcinoma, regional lymph node CA | | PT, CT | | PT/CT  Tumor Localization Whole Body | Radiology | **12.a** | **12.b** | **12.c** |
| 13 | MRI nasopharyngeal carcinoma, regional  lymph node CA | | MR | | MR Neck w and wo IV  Contrast | Neuroradiology | **13.a** | **13.b** | **13.c** |
| 14 | Surgical photos and videos from lymph node dissection | | XC | | Photo - Surgical - Head and  Neck | Otorhinolaryngology | **14.a** | **14.b** | **14.c** |
| 15 | Gross specimen- intake workﬂow, neck lymph nodes | | XC or GM | | Gross Specimen - Head and  Neck | Pathology | **15.a** | **15.b** | **15.c** |
| 16 | Pathology slide scanning workﬂow,  neck lymph nodes | | SM | | Whole Slide - Head and  Neck | Histopathology | **16.a** | **16.b** | **16.c** |
| 17 | Nurse-acquired wound care photographs neck, lymph node incision  evaluation | | XC | | Photo  - Wound - Head and Neck | Wound Care | **17.a** | **17.b** | **17.c** |
| 18 | Mammography | | MG | | Mammo Breast  Screening | Radiology | **18.a** | **18.b** | **18.c** |
| 19 | Medical photographer- acquired dermatology photographs for mole  mapping | | XC | | Photo - Mole Map | Dermatology | **19.a** | **19.b** | **19.c** |
| 20 | Radiology technologist- acquired photograph  swelling right breast | | XC | | Photo  - Breast | Radiology | **20.a** | **20.b** | **20.c** |
| 21 | Tomosynthesis swelling right breast | | Mammo Breast Diagnostic Tomosynthesis  Bilateral | |  | Radiology | **21.a** | **21.b** | **21.c** |
| 22 | Ultrasound swelling  right breast | | US | | US Breast  Right | Radiology | **22.a** | **22.b** | **22.c** |
| 23 | Orbital ultrasound showed a mass posterior to the  left eye | | US | | B-Scan | Ophthalmology | **23.a** | **23.b** | **23.c** |
| 24 | CT mass left eye | | CT | | CT Brain and Orbits  w/Contrast | Radiology | **24.a** | **24.b** | **24.c** |
| 25 | MRI enlarged left optic nerve, r/o optic nerve  glioma | | MR | | MR Optic Nerve | Radiology | **25.a** | **25.b** | **25.c** |
| 26 | Gross specimen-intake workﬂow | | XC or GM | | Gross  Specimen - Eye | Pathology | **26.a** | **26.b** | **26.c** |
| 27 | Pathology slide scanning workﬂow, soft  tissue lobulated lesion in the intraconal optic nerve | | SM | | Whole Slide - Eye | Histopathology | **27.a** | **27.b** | **27.c** |
| 28 | MRI post chemo follow up for Malignant Central Nervous System Primitive Neuroectodermal Tumor (PNET) of the optic nerve, WHO grade  IV, r/o recurrence, r/o metastasis | | MR | | MR Brain and Orbits | Radiology | **28.a** | **28.b** | **28.c** |
| 29 | MRI post chemo follow up for Malignant Central Nervous System Primitive Neuroectodermal Tumor (PNET) of the optic nerve, WHO grade  IV, r/o recurrence, r/o metastasis | | MR | | MR C-  Spine | Radiology | **29.a** | **29.b** | **29.c** |
