# Supplementary material for: Report of the HIMSS-SIIM Enterprise Imaging Community Data Standards Evaluation Workgroup: Anatomic Ontology Assessment
Source: J Imaging Inform Med. 2024 Jun 10;37(6):2709–21. doi: 10.1007/s10278-024-01118-6 (PMC11612098; doi:10.1007/s10278-024-01118-6)
Supplement: Supplementary file 5 — Supplementary file5 (DOCX 29 KB) [file 10278_2024_1118_MOESM5_ESM.docx]

Supplementary Table 3: Assessment of Clinical Use Case for Final Candidate Ontologies

| **1. General Characteristics** | **Weighting factor** | **SNOMED-CT**  **Raw score [weighted score*]** | **ICD-11**  **Raw score [weighted score*]** | **LOINC**  **Raw score [weighted score*]** |
| --- | --- | --- | --- | --- |
| Cost to Organizations RFI Question(s): Q4-Q5 | 3 | 4 [2.25] | 5 [3] | 5 [3] |
| Can terminology be transmitted via a standard coded data type such as with HL7, FHIR, DICOM or IHE XDS? RFI Question(s): Q6-Q10 | 3 | 5 [3] | 5 [3] | 5 [3] |
| Multilingual RFI Question(s): Q11-Q13 | 2 | 5 [2] | 5 [2] | - |
| Hierarchical Structure RFI Question(s): Q14 | 3 | 5 [3] | 5 [3] | - |
| Supports Anatomic Variants RFI Question(s): Q15 | 1 | 5 [1] | 1 [0] | - |
| Includes Laterality RFI Question(s): Q16 | 3 | 5 [3] | 3 [1.5] | - |
| Orientation Included within Nomenclature RFI Question(s): Q17 | 3 | 5 [3] | 5 [3] | - |
| Includes Disorder imaged RFI Question(s): Q18 | 0 | 5 [0] | 5 [0] | - |
| Maps Across Specialties RFI Question(s): Q19 | 3 | 5 [3] | 1 [0] | - |
| **Section 1 average of weighted scores**** |  | **2.53** | **1.94** |  |
| **2. Sustainability** | **Weighting factor** | **SNOMED-CT**  **Raw score [weighted score*]** | **ICD-11**  **Raw score [weighted score*]** | **LOINC**  **Raw score [weighted score*]** |
| Organizational Structure RFI Question(s): Q20 - Q21 | 3 | 5 [3] | 3 [1.5] | - |
| Vendor Usage RFI Question(s): Q22 | 0 | 5 [0] | 5 [0] | - |
| Partnerships RFI Question(s): Q23 - Q24 | 2 | 5 [2] | 1 [0] | - |
| Open-source terminology (vs proprietary) RFI Question(s): Q25 | 1 | 1 [0] | 5 [1] | - |
| Anatomic Region Workgroup Active RFI Question(s): Q26 - Q28 | 2 | 5 [2] | 4 [1.5] | - |
| **Section 2 average of weighted scores**** |  | **1.75** | **1.00** |  |
| **USE CASE 1** | **Weighting factor** | **SNOMED-CT**  **Raw score [weighted score*]** | **ICD-11**  **Raw score [weighted score*]** | **LOINC**  **Raw score [weighted score*]** |
| **USE CASE 1 - Workflow Step 1:** |  |  |  |  |
| Expected Relativity Achieved | 0 | 5 [0] | 5 [0] | - |
| Relationship Mechanism Available | 0 | 5 [0] | 5 [0] | - |
| **USE CASE 1 - Workflow Step 2:** |  |  |  |  |
| Expected Relativity Achieved | 3 | 5 [3] | 5 [3] | - |
| Relationship Mechanism Available | 3 | 5 [3] | 5 [3] | - |
| **USE CASE 1 - Workflow Step 3:** |  |  |  |  |
| Expected Relativity Achieved | 3 | 5 [3] | 5 [3] | - |
| Relationship Mechanism Available | 3 | 5 [3] | 5 [3] | - |
| **USE CASE 1. - Workflow Step 4:** |  |  |  |  |
| Expected Relativity Achieved | 3 | 5 [3] | 1 [0] | - |
| Relationship Mechanism Available | 3 | 5 [3] | 1 [0] | - |
| **USE CASE 1 - Workflow Step 5:** |  |  |  |  |
| Expected Relativity Achieved | 3 | 5 [3] | 1 [0] | - |
| Relationship Mechanism Available | 3 | 5 [3] | 1 [0] | - |
| **USE CASE 1 - Workflow Step 6:** |  |  |  |  |
| Expected Relativity Achieved | 3 | 5 [3] | 1 [0] | - |
| Relationship Mechanism Available | 3 | 5 [3] | 1 [0] | - |
| **USE CASE 1 - Workflow Step 7:** |  |  |  |  |
| Expected Relativity Achieved | 3 | 5 [3] | 1 [0] | - |
| Relationship Mechanism Available | 3 | 5 [3] | 1 [0] | - |
| **USE CASE 1 - Workflow Step 8:** |  |  |  |  |
| Expected Relativity Achieved | 3 | 5 [3] | 1 [0] | - |
| Relationship Mechanism Available | 3 | 5 [3] | 1 [0] | - |
| **USE CASE 1 - Workflow Step 9:** |  |  |  |  |
| Expected Relativity Achieved | 3 | 5 [3] | 1 [0] | - |
| Relationship Mechanism Available | 3 | 5 [3] | 1 [0] | - |
| **USE CASE 1 - Workflow Step 10:** |  |  |  |  |
| Expected Relativity Achieved | 3 | 5 [3] | 1 [0] | - |
| Relationship Mechanism Available | 3 | 5 [3] | 1 [0] | - |
| **USE CASE 1 - Workflow Step 11:** |  |  |  |  |
| Expected Relativity Achieved | 3 | 5 [3] | 1 [0] | - |
| Relationship Mechanism Available | 3 | 5 [3] | 1 [0] | - |
| **USE CASE 1 - Workflow Step 12:** |  |  |  |  |
| Expected Relativity Achieved | 3 | 5 [3] | 1 [0] | - |
| Relationship Mechanism Available | 3 | 5 [3] | 1 [0] | - |
| **USE CASE 1 - Workflow Step 13:** |  |  |  |  |
| Expected Relativity Achieved | 3 | 5 [3] | 1 [0] | - |
| Relationship Mechanism Available | 3 | 5 [3] | 1 [0] | - |
| **USE CASE 1 - Workflow Step 14:** |  |  |  |  |
| Expected Relativity Achieved | 3 | 5 [3] | 1 [0] | - |
| Relationship Mechanism Available | 3 | 5 [3] | 1 [0] | - |
| **USE CASE 1 - Workflow Step 15:** |  |  |  |  |
| Expected Relativity Achieved | 3 | 5 [3] | 1 [0] | - |
| Relationship Mechanism Available | 3 | 5 [3] | 1 [0] | - |
| **USE CASE 1 - Workflow Step 16:** |  |  |  |  |
| Expected Relativity Achieved | 3 | 5 [3] | 1 [0] | - |
| Relationship Mechanism Available | 3 | 5 [3] | 1 [0] | - |
| **USE CASE 1 - Workflow Step 17:** |  |  |  |  |
| Expected Relativity Achieved | 3 | 5 [3] | 1 [0] | - |
| Relationship Mechanism Available | 3 | 5 [3] | 1 [0] | - |
| **USE CASE 1. - Workflow Step 18:** |  |  |  |  |
| Expected Relativity Achieved | 0 | 5 [0] | 5 [0] | - |
| Relationship Mechanism Available | 0 | 5 [0] | 5 [0] | - |
| **USE CASE 1 - Workflow Step 19:** |  |  |  |  |
| Expected Relativity Achieved | 0 | 5 [0] | 1 [0] | - |
| Relationship Mechanism Available | 0 | 5 [0] | 1 [0] | - |
| **USE CASE 1 - Workflow Step 20:** |  |  |  |  |
| Expected Relativity Achieved | 0 | 5 [0] | 5 [0] | - |
| Relationship Mechanism Available | 0 | 5 [0] | 5 [0] | - |
| **USE CASE 1 - Workflow Step 21:** |  |  |  |  |
| Expected Relativity Achieved | 0 | 1 [0] | 5 [0] | - |
| Relationship Mechanism Available | 0 | 1 [0] | 5 [0] | - |
| **USE CASE 1 - Workflow Step 22:** |  |  |  |  |
| Expected Relativity Achieved | 0 | 1 [0] | 5 [0] | - |
| Relationship Mechanism Available | 0 | 1 [0] | 5 [0] | - |
| **Use case 1 average of weighted scores**** |  | **3.00** | **0.38** |  |
| **USE CASE 2** | **Weighting factor** | **SNOMED-CT**  **Raw score [weighted score*]** | **ICD-11**  **Raw score [weighted score*]** | **LOINC**  **Raw score [weighted score*]** |
| **USE CASE 2 - Workflow Step 23:** |  |  |  |  |
| Expected Relativity Achieved | 0 | 5 [0] | 5 [0] | - |
| Relationship Mechanism Available | 0 | 5 [0] | 5 [0] | - |
| **USE CASE 2 - Workflow Step 24:** |  |  |  |  |
| Expected Relativity Achieved | 3 | 5 [3] | 5 [3] | - |
| Relationship Mechanism Available | 3 | 5 [3] | 5 [3] | - |
| **USE CASE 2 - Workflow Step 25:** |  |  |  |  |
| Expected Relativity Achieved | 3 | 5 [3] | 5 [3] | - |
| Relationship Mechanism Available | 3 | 5 [3] | 5 [3] | - |
| **USE CASE 2 - Workflow Step 26:** |  |  |  |  |
| Expected Relativity Achieved | 3 | 5 [3] | 5 [3] | - |
| Relationship Mechanism Available | 3 | 5 [3] | 5 [3] | - |
| **USE CASE 2 - Workflow Step 27:** |  |  |  |  |
| Expected Relativity Achieved | 3 | 5 [3] | 1 [0] | - |
| Relationship Mechanism Available | 3 | 5 [3] | 1 [0] | - |
| **USE CASE 2 - Workflow Step 28:** |  |  |  |  |
| Expected Relativity Achieved | 3 | 5 [3] | 1 [0] | - |
| Relationship Mechanism Available | 3 | 5 [3] | 1 [0] | - |
| **USE CASE 2 - Workflow Step 29:** |  |  |  |  |
| Expected Relativity Achieved | 3 | 5 [3] | 1 [0] | - |
| Relationship Mechanism Available | 3 | 5 [3] | 1 [0] | - |
| **Use case 2 average of weighted scores**** |  | **3.00** | **1.50** |  |
| **Sum of average weighted scores***** |  | **10.28** | **4.81** |  |

* Weighted score = ((Raw score – 1)/4) * weighting factor

** Average of weighted scores = mean of all weighted scores for a section where the weighting factor is > 0

*** Sum of average weighted scores = [Section 1 average of weighted scores] + [Section 2 average of weighted scores] + [Use case 1 average of weighted scores] + [Use case 2 average of weighted score]
